# Effect of *Triphala* mouthwash on the caries status

**DOI:** 10.4103/0974-7788.64413

**Published:** 2010

**Authors:** Shobha Tandon, Kunal Gupta, Sugandhi Rao, K. J. Malagi

**Affiliations:** 1 Former Post-graduate Student, Department of Pedodontics, Manipal College of Dental Sciences, Manipal - 576 104, India; 2 Professor, Department of Microbiology, Kasturba Medical College, Manipal - 576 104, India; 3 Associate Professor, Department of Ayurveda, Kasturba Medical College, Manipal - 576 104, India

**Keywords:** Dental caries, *Triphala*

## Abstract

Nearly 60–70% of the child Indian population suffers from dental caries. Mouth rinsing is the most cost effective method of preventing dental caries. '*Triphala*' has been a classic Ayurveda remedy, probably the best known among all Ayurvedic compounds. This study was conducted on 1501 students in the age group of 8-12 years with the aim of determining the effect of *Triphala* mouthwash on prevention of dental caries (manifest caries) as well as incipient carious lesions, and also comparing the effect of *Triphala* and chlorhexidine mouthwashes. The incipient caries was recorded at 3, 6, 9 months intervals and manifest caries at 9 months interval. No significant increase in the DMFS scores was found at the end of 9 months. Also, there was no significant increase in the incipient caries score towards the conclusion of the study. It was concluded that there was no significant difference between the *Triphala* and the chlorhexidine mouthwashes.

## INTRODUCTION

Dental caries and gingivitis are the most prevalent diseases of mankind and these diseases are multifactorial in origin. In India, nearly 60–70% of the child population is affected by dental caries.[1] The geostrata of India is such that majority of the population lives in the rural areas where the accessibility to dental treatment is quite limited since the majority of dentists reside in urban areas. There is a lot of cost as well as manpower involved in the treatment of these diseases. It is indeed a challenging and tough task to reduce the occurrence and severity of these problems.

Prevention, including the use of chemical therapies, is more cost effective as the patient shifts from a high-risk to a low-risk level. Recall appointments can subsequently be extended and more conservative prevention treatments are warranted. Over an extended treatment period, the cost for the preservative dentistry option is comparable to and perhaps less than the cost of placing and replacing dental restorations.[2]

Mouth rinsing for the prevention of dental caries in children and adolescents was established as a mass prophylactic method in the 1960s and has shown an average efficacy of caries reduction between 20 and 50%. Mouth rinsing solutions have been combined with antiplaque agents like chlorhexidine and other agents, which can improve the caries preventive effect in high caries risk patients. '*Triphala*' has been described as a classic Ayurveda remedy, probably the best known among all Ayurvedic compounds. There is little literature available on its beneficial effects on oral cavity and on oral hygiene.

This study was hence planned to evaluate clinically the efficacy of *Triphala* mouthwash in preventing the occurrence of Incipient lesions or manifest lesions of dental caries. We also wished to compare the effect of *Triphala* mouthwash with commercially available chlorhexidine mouthwash.

## MATERIALS AND METHODS

This study was conducted in the Department of Pedodontics and Preventive Dentistry, College of Dental Surgery, Manipal, in collaboration with Department of Ayurveda and Department of Microbiology, Kasturba Medical College, MAHE after obtaining Ethics Committee Permission. A total of 1501 students in the age group of 8–12 years, belonging to classes 4^th^ to 6^th^ were the subjects for this study and a total of eight schools around Manipal were included in this study.

Materials used for recording indices were mouth mirror, explorer, tweezers and chip syringe; for oral prophylaxis - hand scalers, disclosing solution, polishing paste, cup and brush, portable micromotor with handpiece.

Before the commencement of the study, an informed consent from the principal of the school as well as the parents of the students participating in the study was obtained.

The students were selected keeping in mind that they had almost similar socioeconomic status, dietary habits, oral hygiene methods, oral hygiene status and KAP status.

Children had a minimum of 1–2 established carious lesions.

The subjects were divided into three groups by block randomization: Group I (*n* = 514) - using *Triphala* mouthwash (0.6%), Group II (*n* = 495) – using chlorhexidine mouthwash (0.1%) [Positive control] and Group III (*n* = 492) – using Distilled water [Negative control].

The schools were distributed in such a manner so that there was no intermingling within the students of different groups. The group allotment was done in such a manner that the investigators and teachers, who were trained to administer the mouthwash, did not know the content of the bottles. Also, the subjects were not told about the content of mouthwash to make this study a complete double-blind clinical trial.

The caries status was assessed using the DMFS index[3] (DMF is index for recording dental caries in permanant teeth whereas dmf is index recording dental caries in primary / milk teeth) and incipient caries using the DSi index as suggested by Bjarnason, Kohler and Ranggard.[4]

Dental caries for DMFS index was diagnosed based on the following criteria:

1. The lesion was clinically visible and obvious.
2. The explorer tip could penetrate deep into soft yielding material.
3. There was discoloration or loss of translucency typical of undermined or demineralized enamel.
4. The explorer tip got caught in a pit or fissure or resisted removal after moderate to firm pressure on insertion.

For posterior teeth five surfaces were examined and for anterior teeth four surfaces were examined.

Incipient caries for DSi index was diagnosed visually under proper light after drying the tooth surface with cotton. It appeared as white chalky area when dried and was not visible when moistened with saliva.

Approximately 0.6% *Triphala* mouthwash, 0.1% chlorhexidine mouthwash and distilled water were prepared in the pharmacy manufacturing unit of the hospital. All solutions were made of identical colors to eliminate bias. The bottles were then coded and then at the end of the study, the decoding was done.

The indices were recorded before commencement of the study. After collecting the baseline data, plaque and gingivitis scores were brought to zero. Mouth rinsing was started on the day of performing the oral prophylaxis. The teachers were educated and trained in the use of mouthwash so that the children , under the supervision of the teachers, could use the mouthwash. Each of the groups used the respective mouthwash, as a daily, supervised rinse after lunch in the afternoon. The children were advised not to eat or rinse for the next 30 minutes. They were instructed to carry home the mouthwash bottles on weekends and during vacations.

Ten milliliters of mouthwashes were dispensed at one time. The mouthwash was swished in all quadrants of the mouth for a period of two minutes.

The Dsi (incipient caries) were done at 3, 6 and 9 months intervals from baseline and the caries assessment was done at 9 months interval from baseline.

For intra-group comparison of the paired sample, *t*-test was applied, while for the inter-group comparison independent *t*-test was applied. All the tests were carried out using SPSS package in the computer.

## RESULTS

### Attrition of the data

There was attrition of the sample in all the three groups and the number of subjects at the end of the study was 495 in group A, 475 in group B and 461 in group C. The percentage of attrition in Group A, B and C was 3.69, 4.04 and 6.30%, respectively. Overall attrition of the entire sample was 4.66%.

### Caries score

#### Baseline

The mean DMFS score in group I was 0.87 ± 1.80, 1.18 ± 1.95 in group II and 1.26 ± 2.09 in group III (control). The mean dmfs scores were 7.98 ± 8.86, 8.12 ± 8.11 and 6.26 ± 7.56 in groups I, II and III, respectively [Table 1].

**Table 1:** Mean DMFS and dmfs scores before starting of mouthwash regimen

Groups	DMFS ± SD	dmfs ± SD
Group I	0.87 ± 1.80	7.98 ± 8.86
Group II	1.18 ± 1.95	8.12 ± 8.11
Group III	1.26 ± 2.09	6.26 ± 7.56

#### Nine months

The mean DMFS score at the conclusion of the study in group I was 1.22 ± 2.04, 1.36 ± 2.13 in group II and 1.91 ± 2.66 in group III (control). The mean dmfs scores were 6.60 ± 7.66, 7.75 ± 7.90 and 5.46 ± 7.73 in groups I, II and III, respectively [Table 2].

**Table 2:** Mean DMFS and dmfs scores at 9 months mean ± standard deviation

Groups	DMFS	dmfs
Group I	1.22 ± 2.04	6.60 ± 7.66
Group II	1.36 ± 2.13	7.75 ± 7.90
Group III	1.91 ± 2.66	5.46 ± 7.73

#### Incipient caries

Incipient caries score at baseline in group I was 0.75 ± 1.18, in group II it was 0.53 ± 0.87 and 0.27 ± 0.54 in group III [Table 3].

**Table 3:** Mean DSi scores before starting of mouthwash regimen

Groups	DSi scores ± SD
Group I	0.75 ± 1.18
Group II	0.53 ± 0.87
Group III	0.27 ± 0.54

#### Three months

The mean incipient caries scores of group I (*Triphala*) and group II (chlorhexidine) at 3 months interval was 0.71 + 0.89 and 0.48 ± 0.86 respectively. In the control group the incipient caries score was 0.49 ± 0.85 [Table 4].

**Table 4:** Mean DSi scores at various time intervals mean ± standard deviation

Groups	3 months	6 months	9 months
Group I	0.71 ± 0.89	0.68 ± 0.86	0.69 ± 0.89
Group II	0.48 ± 0.86	0.46 ± 0.84	0.54 ± 0.90
Group III	0.49 ± 0.85	0.70 ± 0.91	0.78 ± 0.94

Intragroup Comparison Of Incipient Caries Scores

#### Six months

The mean incipient caries scores of groups I, II and III at 6 months interval were 0.68 ± 0.86, 0.46 ± 0.84 and 0.70 ± 0.91, respectively [Table 4].

#### Nine months

The mean incipient caries scores at 9 months interval of groups I, II and III were 0.69 ± 0.89, 0.54 ± 0.90 and 0.78 ± 0.94, respectively [Table 4].

## DISCUSSION

This study was conducted in the Department of Pedodontics and Preventive Dentistry, in collaboration with the Department of Microbiology, Kasturba Medical College, Manipal over a period of 9 months. The study was a double-blind controlled clinical trial. Overholser[5] had proposed that long-term clinical studies to evaluate the effectiveness of antimicrobial mouth rinses should be double blind and last for a minimum of 6 months.

Eight schools were chosen from areas around Manipal. A total of 1501 children having the same socioeconomic status and oral hygiene practice, in the age group of 8-12–years were chosen for the study. These students were divided into three groups by block randomization. Group I (514 students) used the *Triphala* mouthwash (0.6%), in Group II (495 students) chlorhexidine (0.1%) mouthwash was provided and in Group III (492 students) distilled water was used.

At the end of the study, there was an overall attrition of the sample by 4.66%. The individual group attrition was 3.69% in Group A, 4.04% in Group B and 6.30% in Group C. This attrition was because many students changed schools after completion of an annual session, which had fallen during the period of the study. This attrition of 4.66% was not considered to be significant in this study as it had a large sample size.

The division into groups was done in such a way that there was no intermingling of students from different groups. This was done to prevent any discussion amongst the students on the type and taste of the mouthwash, they are using.

The students did not discontinue with their routine oral hygiene practices. Similar study design was followed in a six weeks trial conducted by Axelsson,[6] where the subjects continued to exercise their regular non-supervised, self-performed plaque control measures.

The effect of *Triphala* extract against *Streptococcus mutans* and *Lactobacillus* was determined by agar well diffusion method. The zone of inhibition obtained with various concentrations of the extract was observed after 24 h of incubation at 37°C. It was found that 0.6% of *Triphala* extract showed maximum effect against *S. mutans* and *Lactobacillus*.

Ernst[7] stated that the increase in concentration of chlorhexidine from 0.1 to 0.2% provided no clinical advantages or disadvantages. Hence, in this study 0.1% concentration was used since the mouthwash had to be used for a longer period of time. Addy[8] also stated that 0.1% formulation produced less staining, particularly when diluted.

Group III served as the control group and was included in the study to rule out any effect, which could be due to the mechanical effect of rinsing.

The efficacy of the mouthwash was tested against Caries,[3] Incipient caries.[4] Following this, the plaque and gingivitis scores were brought down to zero by means of oral prophylaxis. After this mouthwash was administered, the indices were recorded at 3, 6, 9 months intervals except for DMFS/dmfs which were recorded only after 9 months. These indices were used as they are simple and are mostly employed in controlled clinical trials of preventive and therapeutic agents.

Children were instructed to rinse their mouth with 10 ml of solution for a period of 1 min after lunch and then were told not to rinse their mouth with water or drink anything for half an hour. Similar amount and duration of mouthwash administration was followed in a study conducted by Axelsson.[6]

The chlorhexidine preparation did not contain alcohol. Leyes, Horrajo and Garcia[9] stated that the alcohol-free rinse was as effective as one containing alcohol in controlling plaque and reducing gingival inflammation.

### Incipient caries [Figure 1]

**Figure 1:**
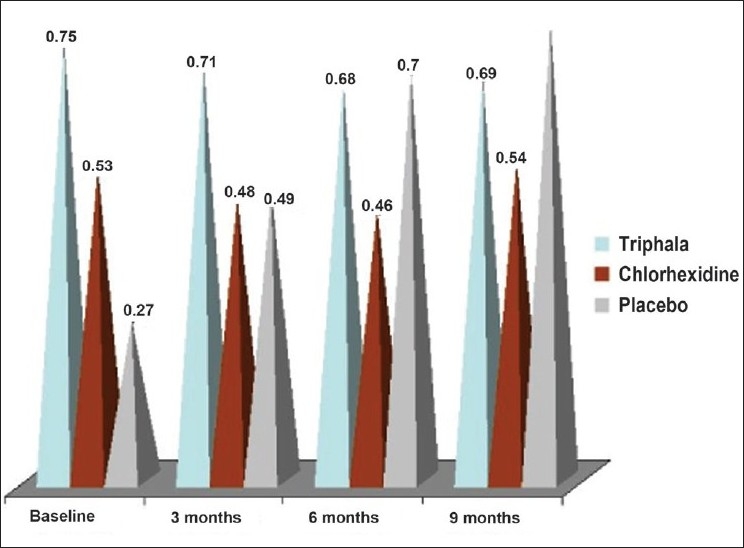
Mean incipient caries scores

In group I, there was a nonsignificant decrease in the incipient caries from the baseline to the end of the third month (0.75 ± 1.18 to 0.71 ± 0.89). Thereafter, there was a decrease in the incipient caries score to 0.68 ± 0.86 by the end of six months and then a very marginal increase till the conclusion of the study (0.69 ± 0.89) [Tables 3 and 4]. This observation indicated that a few of the incipient lesions transformed into manifested caries lesions, when there was a decrease in the score and that a few of the incipient lesions newly developed when there is an increase in the incipient caries score. The difference in the incipient caries in all the intervals was nonsignificant (*P* > 0.05) [Table 5]. Thus, suggesting that the *Triphala* mouthwash prevents any significant change in the incipient caries score.

**Table 5:** Intragroup comparison of incipient caries scores in group I

Incipient caries	Mean difference ± SD	*P* value
I0 vs I3	0.0465 ± 1.0872	0.342
I0 vs I6	0.0748 ± 1.2024	0.167
I0 vs I9	0.0727 ± 1.0603	0.128
I3 vs I6	0.0283 ± 0.6129	0.305
I3 vs I9	0.0263 ± 0.9530	0.540
I6 vs I9	-0.0202 ± 0.9597	0.963

I0 = Baseline Incipient Caries score, I3 = Incipient Caries score at 3 months, I6 = Incipient Caries score at 6 months, I9 = Incipient Caries score at 9 months

In group II, there was a decrease in the incipient caries score from baseline till the end of six months (0.53 ± 0.87 - baseline; 0.48 ± 0.86 - 3 months; 0.46 ± 0.84 - 6 months) [Tables 3 and 4]. After that, there was an increase in the score to 0.54 ± 0.90, which was statistically significant (*P* = 0.014) [Table 6]. However, on comparing the baseline scores to the nine months scores, it is evident that there is no significant change in the incipient caries score. This again suggests that the chlorhexidine mouthwash prevents significant changes in the incipient caries. Loe[10] used a 0.2% chlorhexidine mouthrinse to prevent the development of white spot lesions. Vonder Fehr,[11] however, stated that it had a little role in preventing dental caries. Similar trend was observed in our study.

**Table 6:** Intragroup comparison of incipient caries scores in group II

Incipient caries	Mean difference ± SD	*P* value
I0 vs I3	0.0505 ± 0.6411	0.086
I0 vs I6	0.0632 ± 0.6876	0.046
I0 vs I9	-0.0189 ± 0.5752	0.473
I3 vs I6	0.0126 ± 0.3786	0.467
I3 vs I9	-0.0695 ± 0.7529	0.045
I6 vs I9	-0.08211 ± 0.7289	0.014

In group III (distilled water), it was found that there was a significant increase in the incipient caries from the baseline (0.27 ± 0.54) to all the intervals [Tables 3, 4 and 7]. This suggests that the distilled water is not capable of preventing the initiation of incipient lesions.

**Table 7:** Intragroup comparison of incipient caries scores in group III

Incipient caries	Mean difference ± SD	*P* value
I0 vs I3	–0.2104 ± 0.8755	0.001
I0 vs I6	–0.4295 ± 0.9702	0.001
I0 vs I9	–0.5098 ± 1.0604	0.007
I3 vs I6	–0.2191 ± 0.6929	0.001
I3 vs I9	–0.2993 ± 0.8928	0.001
I6 vs I9	–0.0803 ± 0.8871	0.053

Intragroup Comparison Of DMFS/Dmfs scores

### DMFS scores [Figure 2]

**Figure 2:**
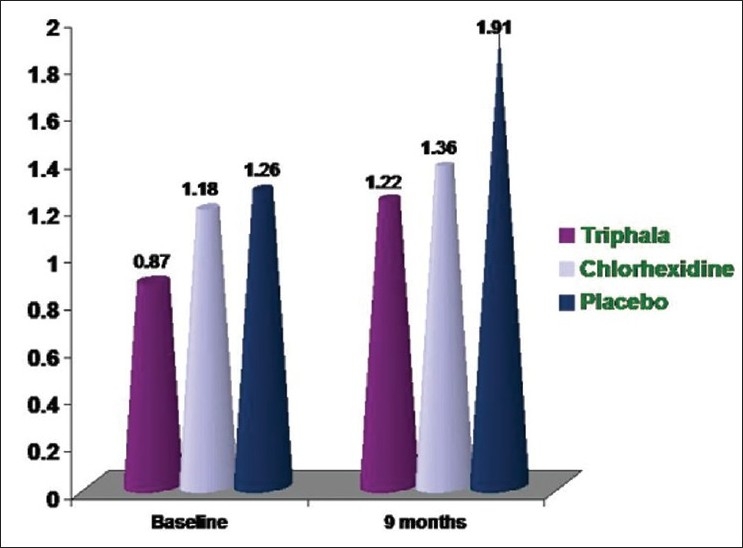
Mean DMFS scores

In group I (*Triphala*), there was an increase in the DMFS scores from 0.87 ± 1.80 [Table 1] to 1.22 ± 2.04 [Table 2] by the end of 9 months. This increase was however found to be not significant (*P* = 0.145) [Table 8]. This suggests that the *Triphala* mouthwash prevents any significant increase in the caries status of the permanent dentition.

**Table 8:** Intragroup comparison of DMFS/dmfs scores in group I

DMFS/dmfs	Mean difference ± SD	*P* value
D0 vs D9	–3.495 ± 4.226	0.145
d0 vs d9	1.385 ± 4.994	0.238

D0 = Baseline DMFS score, D9 = DMFS score at 9 months, d0 = Baseline dmfs score, d9 = dmfs score at 9 months

In a study conducted by Aman,[12] it was found that a cupful of pure amla juice (a constituent of *Triphala*) and honey was useful for preventing dental caries.

In group II (chlorhexidine), there was an increase in the DMFS scores from 1.18 ± 1.95 [Table 1] to 1.36 ± 2.13 [Table 2] by the end of 9 months. This increase again was not found to be significant, as was observed with *Triphala* (*P* = 0.134) [Table 9]. Okada[13] observed that after using chlorhexidine, there was a reduction in the caries status in only four out of eight students, two showed trend towards less caries and two showed no benefit from the mouthwash. This partially supports our observation where there is a nonsignificant increase in the caries.

**Table 9:** Intragroup comparison of DMFS/dmfs scores in group II

DMFS/dmfs	Mean difference ± SD	*P* value
D0 vs D9	–0.1832 ± 4.825	0.134
d0 vs d9	0.3642 ± 4.924	0.108

In group III (distilled water) there was an increase in the DMFS scores (1.26 ± 2.09 [Table 1] to 1.91 ± 2.66 [Table 2]) but contrary to the other two groups. This increase however, was found to be highly significant (*P* = 0.001) [Table 10].

**Table 10:** Intragroup comparison of DMFS/dmfs scores in group III

DMFS/dmfs	Mean difference ± SD	*P* value
D0 vs D9	–0.6616 ± 1.347	0.001
d0 vs d9	0.7983 ± 5.603	0.002

Intergroup comparison between the groups II and I [Table 11] revealed that there was no significant difference between them by the end of 9 months suggesting that the *Triphala* and chlorhexidine mouthwash are comparable in preventing any significant increase in the caries status in a permanent dentition.

**Table 11:** Intergroup comparison of DMFS scores - Post intervention (9 months)

Mouth wash	Mean ± SD	Groups		
		Group I	Group Il	Group III
Group I	1.22 ± 2.04		*P* = 0.283	*P = 0.000
Group II	1.36 ± 2.13			*P = 0.001
Group III	1.91 ± 2.66			

Where

* indicates groups significantly diff erent at *P* < 0.05

Fine[14] stated that chlorhexidine mouth rinses effective against *S. mutans* also have a role in inhibiting dental caries. A study was conducted by Jalil,[15] who stated that there is a significant trend of increasing decayed, missing, filled surfaces (DMFS) with increasing *S. mutans* counts.

However in our study, it can be observed that although there is an increase in the DMFS scores as the *S. mutans* counts reduce but this is not significant. Another study[16,17] also supports our observation, where it was found that high *S. mutans* counts are not invariably associated with caries.

### DMFS scores [Figure 3]

**Figure 3:**
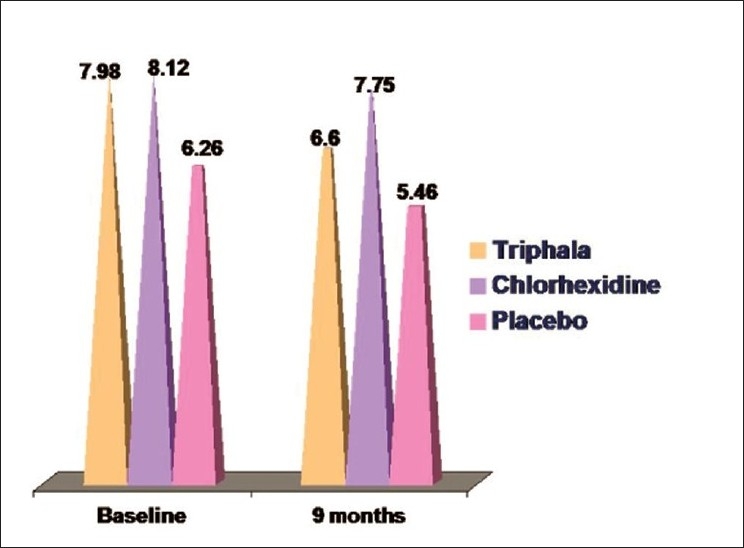
Mean dmfs scores

A decrease in the dmfs scores were observed in all the groups as a number of teeth, which had caries at the baseline, were exfoliated by 9 months, thereby reducing the caries status.

In group I (*Triphala*), there was a decrease in the caries score from 7.98 ± 8.86 [Table 1] at the baseline to 6.60 ± 7.66 [Table 2] by the end of 9 months. This decrease was found to be not significant (*P* = 0.238) [Table 8].

In group II (chlorhexidine), there was again a decrease in the caries status from 8.12 ± 8.11 [Table 5] to 7.75 ± 7.90 [Table 2] by the end of nine months. This decrease was found to be non-significant (*P* = 0.108) [Table 9].

In group III (distilled water), there was a decrease in the dmfs scores from 6.26 ± 7.56 [Table 1] to 5.46 ± 7.73 [Table 2] by the end of nine months. This decrease however was found to be highly significant (*P* = 0.002) [Table 10].

Intergroup comparison shows that there was a significant difference (*P* < 0.05) between all the groups by the end of nine months [Table 12].

**Table 12:** Intergroup comparison of dmfs scores - Post intervention (9 months)

Mouth wash	Mean ± SD	Groups		
		Group I	Group II	Group III
Group I	6.60 ± 7.66		*P =0.021	*P =0.023
Group II	7.75 ± 7.90			*P =0.001
Group III	5.46 ± 7.73			

Where

* indicates groups significantly diff erent at *P* < 0.05

Since the deciduous teeth exfoliate during the period of the study, they cannot be a true indicator of the effect of mouthwash on the caries status of the deciduous teeth.

## CONCLUSION

This study was carried on 1501 students in age group of 8–12 years. The overall attrition of the data was 4.66%. The study was conducted with the aim of eliciting the effect of *Triphala* on the oral health status with regards to the caries scores. Recording of caries (manifest and incipient) was done. A thorough oral prophylaxis was done to bring plaque scores to zero and all the groups used the respective mouthwashes (*Triphala* mouthwash, chlorhexidine mouthwash and distilled water) as a supervised rinse. The incipient caries was recorded at 3, 6, 9 months intervals and manifest caries at 9 months interval. The data collected was subjected to statistical analysis.

The following conclusions were drawn from the present study:

1. With the use of *Triphala*, although there was an increase in the DMFS scores, it was not significant. The trend in the caries status was comparable to that of chlorhexidine. dmfs scores could not indicate the efficacy of mouthwash as this was a long-term study of nine months and a few of the carious teeth recorded at baseline were exfoliated at the conclusion of the study. However, no new manifested carious lesions were observed.
2. Although there was a reduction in the incipient caries scores, the role of mouthwash in the process of remineralization could not be established. There was no significant increase in the incipient caries score towards the conclusion of the study suggesting that *Triphala* mouthwash has a role to play in preventing the development of incipient lesions.
3. In group III where distilled water was administered, the caries status was found to increase significantly.
4. The cost of *Triphala* mouthwash is much less compared to commercially available chlorhexidine mouthwash. Being an Ayurvedic product, it has no known side effects compared to chlorhexidine and hence is safe for use over a long period of time.

Thus, overall comparison showed that 0.6% *Triphala* and 0.1% chlorhexidine are comparable in preventing a significant increase in the caries status. This clinical trial of longer duration with a larger sample size should play a vital role in commercialization of *Triphala* mouthwash.

Given the increasing interest in Ayurveda and the enormous power to two contemporary approaches – evidence-based clinical practice and modern biology – the time is ripe to reformulate our approach to the practice, research and training in Ayurveda.
